# Rock substrate rather than black stain alterations drives microbial community structure in the passage of Lascaux Cave

**DOI:** 10.1186/s40168-018-0599-9

**Published:** 2018-12-05

**Authors:** Lise Alonso, Charline Creuzé-des-Châtelliers, Théo Trabac, Audrey Dubost, Yvan Moënne-Loccoz, Thomas Pommier

**Affiliations:** Univ Lyon, Université Claude Bernard Lyon 1, CNRS, INRA, VetAgro Sup, UMR5557/1418 Ecologie Microbienne, 69622 Villeurbanne, France

**Keywords:** Lascaux Cave, Microbial degradation, Bacterial-fungal co-occurrence, Microbial community

## Abstract

**Background:**

The World-famous UNESCO heritage from the Paleolithic human society, Lascaux Cave (France), has endeavored intense microclimatic perturbations, in part due to high touristic pressure. These perturbations have resulted in numerous disturbances of the cave ecosystem, including on its microbial compartment, which resulted in the formation of black stains especially on the rock faces of the passage. We investigated the cave microbiome in this part of Lascaux by sampling three mineral substrates (soil, banks, and inclined planes) on and outside stains to assess current cave microbial assemblage and explore the possibility that pigmented microorganisms involved in stain development occur as microbial consortia.

**Methods:**

Microbial abundance and diversity were assessed by means of quantitative PCR and high-throughput sequencing (Illumina MiSeq) of several DNA and cDNA taxonomic markers. Five sampling campaigns were carried out during winter and summer to embrace potential seasonal effect in this somewhat stable environment (based on measurements of temperature and CO_2_ concentration).

**Results:**

While the season or type of mineral substrate did not affect the abundances of bacteria and micro-eukaryotes on or outside stains, mineral substrate rather than stain presence appears to be the most significant factor determining microbial diversity and structuring microbial community, regardless of whether DNA or cDNA markers were considered. A phylogenetic signal was also detected in relation to substrate types, presence of stains but not with season among the OTUs common to the three substrates. Co-occurrence network analyses showed that most bacterial and fungal interactions were positive regardless of the factor tested (season, substrate, or stain), but these networks varied according to ecological conditions and time. Microorganisms known to harbor pigmentation ability were well established inside but also outside black stains, which may be prerequisite for subsequent stain formation.

**Conclusions:**

This first high throughput sequencing performed in Lascaux Cave showed that black stains were secondary to mineral substrate in determining microbiome community structure, regardless of whether total or transcriptionally active bacterial and micro-eukaryotic communities were considered. These results revealed the potential for new stain formation and highlight the need for careful microbiome management to avoid further cave wall degradation.

**Electronic supplementary material:**

The online version of this article (10.1186/s40168-018-0599-9) contains supplementary material, which is available to authorized users.

## Background

Lascaux cave is famous for its paintings dating from the Upper Paleolithic (ca. 18,000 AD) [1]. Since its discovery in 1940, this jewel of humanity (recognized as such by the UNESCO in 1979) suffered several ecosystem disturbances mostly associated with the development of tourism. The visits, which reached up to ~ 1800 visitors per day in the 1960s, strongly modified the microclimatic conditions of the cave in terms of temperature, light conditions, and CO_2_ concentration [2]. They resulted in several microbe-related “diseases,” materialized by various developments of stains on the walls, i.e., green stains, later on white stains and more recently black stains [3, 4].

The successive microbial diseases were treated with the application of biocides and antibiotics, which promoted the development of resistant microorganisms [5]. In other environments, similar treatments have resulted in presence of adapted bacteria, e.g., *Pseudomonas aeruginosa* and *Pseudomonas stutzeri* and drastic changes in microbial community structures [6, 7]. For instance in fish farm sediments, the number of bacteria declined by 50–67% due to the application of antibiotics [8], whereas  in agricultural soils application of sulfonamide antibiotics could affect bacterial community structure [9]. Similarly, fungi are often sensitive to antifungal substances that dramatically constrain their community structure to the few resistant strains [10]. However, fungi are generally more resistant to biocides than bacteria [11], which is mostly due to their composition of outer cell layers with chitin. Although little is known about the responses to fungicides and antibiotic treatments in cave environments, it is thought that such treatments had altered the microbial community of Lascaux Cave [5]. However, since its pristine microbial community is not known, disentangling the relative impacts of the treatments from previous human activity is highly challenging.

**Fig. 1:**
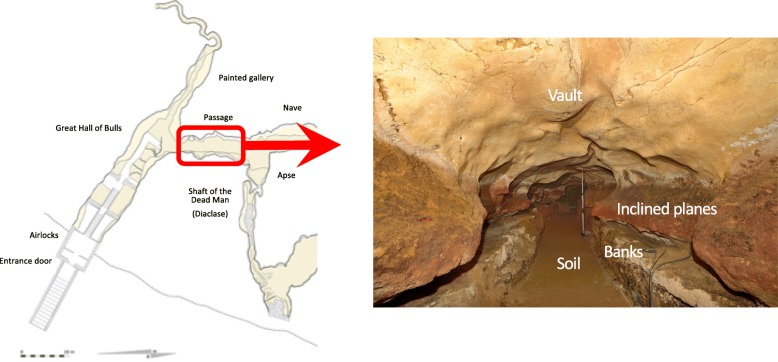
Map of Lascaux Cave with location of the passage (source: MCC-CNP) and internal structure of the passage with the three mineral substrates studied (soil, banks, inclined planes) (source: S. Konik, *Centre National de la Préhistoire*; photograph taken on 29 September 2017)

Although several studies have shown that microbial community is abundant and diverse in caves despite the fact that caves are oligotrophic environments [12–14], the ecology in Lascaux cave may not follow similar patterns, mostly due to the dense touristic pressure and the recurrent antibiotic treatments described above. Indeed, in cave environments poor in nutrients, microorganisms are thought to cooperate to optimize resource utilization rather than competing [15]. In Lascaux cave, the nutrient levels may sustain high microbial growth with little limitations. Some bacterial phyla (*Proteobacteria*, *Actinobacteria*) are usually predominant in non-anthropized caves, but the overall microbial composition is specific to each cave and related to the type of cave (limestone, lava, ice) and their environmental conditions (pH, availability of nutrients, humidity) (e.g., [16, 17]). Lascaux Cave is a limestone cave with relative humidity near 100% and a mean annual temperature of 12.6 °C. The passage is a central node of communication between the 12 chambers of Lascaux Cave and harbors heterogeneous mineral substrates, i.e., a floor of beaten earth consisting of local calcareous sand, a bank of clay deposits, near-vertical walls (termed inclined planes) of limestone, and a vault where the limestone had been covered by a calcite layer (Fig. 1). Since 2006, black stains appeared on the vault and the inclined planes, and then propagated on other mineral substrates (i.e., the banks) and other chambers, threatening paintings [3]. A previous culture-based microbiological study of this part of the cave after chemical treatments provided a large number of isolates, among which the most abundant bacteria belonged to *Ralstonia* and *Pseudomonas* genus and the most abundant fungi to *Exophiala* genus and *Fusarium solani* species [5]. Because these genera are rarely predominant in natural caves, it is thought that they have been selected by chemical treatments [5]. Some of the fungal taxa selected include strains with pigmentation potential, and black fungal isolates, i.e., from *Ochroconis lascauxensis*, *Exophiala moniliae*, and *Acremonium nepalense* have been obtained from the passage [18]. Some of them, e.g., *Exophiala salmonis*, have also been found on cave wall samples or in extreme environments, e.g., desert areas [19]. Since fungal melanin (black coloration) can confer resistance to many types of environmental stress [20], these pigments may be an important asset to resist to chemical treatments in caves. In the passage, black stains cover a minority of cave wall surfaces, and the vast majority does not display surface alterations. The occurrence of black stains most likely results from spatially localized conditions promoting particular development or physiology of pigmented microorganisms, and the formation of black stains itself should lead, in turn, to specific surface conditions. On this basis, we hypothesized that black stains could feature similar microbial communities even if they are located on different mineral substrates. If this hypothesis holds true, it would mean that the presence of stain is as influential as the type of mineral substrate. This question was the focus of the present investigation.

The objective of this work was to assess the relative importance of mineral substrate, presence of black stains and temporal variations in determining microbial community structure. To this end, we applied high throughput sequencing of bacterial, eukaryotic, and fungal taxonomic markers amplified from the DNA of 112 samples collected during 5 campaigns between 2014 and 2016 in the passage. This aimed also at describing the microbial consortia associated with pigmented microorganisms in Lascaux Cave, and better understanding microbial successions in stable environments such as continental caves. We further compared these DNA-based microbial patterns with those obtained from RNA extracted from a selection of samples to analyze how the active community responded to the same environmental variables. Finally, we investigated whether co-occurrence networks were driven by environmental pressures and examined the associated phylogenetic patterns.

## Methods

### Sampling site and environmental data measurements

Lascaux Cave is located in Dordogne in South-West France (N 45° 03′ 13″ and E 1° 10′ 12″) and has been closed for touristic visits since 1963 due to cave wall degradations. Since then, human presence is highly restricted and restrained to scientific campaigns and official visits. The passage in Lascaux Cave was selected for sampling due to its central location and because black stains occurred early in this part of the cave before similar stains also formed in neighboring chambers of the cave. The passage spans 16.6 m in length and 1.6 m in width. Temperature and CO_2_ concentration are monitored continuously with sensors, and records are processed every minute using an AMR WinControl (Akrobit®) software. For this study, we used the monthly average temperature and CO_2_ concentration during the month preceding sampling. The passage presents four main zones (from bottom to top: soil, banks, inclined planes, vault) that are defined according to their mineral substrate and topography: beaten-earth soil (calcareous sand), banks (clay sediments), inclined planes (limestone), and vault (limestone covered by calcite).

To avoid degradation of Paleolithic art, samples were only taken on the soil, the banks, and art-free parts of the inclined planes, but not on the vault (too fragile). Five sampling campaigns were performed in December 2014, June–July 2015, January 2016, May–June 2016, and December 2016 to study the present microbial community (DNA analyses). In January 2016, supplementary samples were also taken to assess the active microbial community (RNA analyses). In each campaign, samples were taken in four different days spread over a 16-day period to limit the impact of human presence, in accordance with cave rules and regulations. For each mineral substrate and each sampling campaign, 3–6 samples of ~ 50 mg material were taken on (when present) and outside black stains using sterile scalpels. In total, 136 samples were collected for this study. To avoid community changes during storage, the samples were immediately placed in liquid nitrogen and transferred later to − 80 °C until DNA and RNA extraction. Due to the limited amount of available material for each sample, RNA and DNA could not be extracted simultaneously.

### Nucleic acids extraction, quantitative PCRs, and high-throughput sequencing

DNA extraction was performed using the FastDNA SPIN Kit for Soil (MP Biomedicals, Illkirch, France), following the manufacturer’s instructions and adapted to low amounts of sample. The elution step was achieved using two volumes of 50 μl elution buffer for each sample. RNA extraction from cave samples was performed using ZR Soil/Fecal RNA MicroPrep kit (Zymo Research, Irvine, CA), following the manufacturer’s instructions. The DNase treatment was performed during extraction with DNase I Set (Zymo Research). The resulting DNA and RNA concentrations were quantified using the Qubit RNA BR Assay Kit (Thermo Fisher Scientific, Eugene, OR) following the manufacturer’s instructions. DNA and RNA extracts were stored at − 80 °C until further analysis. The reverse transcription of RNA extracts into complementary DNA (cDNA) was carried out by Fasteris compagny (Geneva, Switzerland) before high-throughput sequencing (Illumina MiSeq).

To assess the numbers of bacterial 16S rRNA genes and micro-eukaryotic 18S rRNA genes, quantitative PCR (qPCR) was performed on 127 samples (52 on stains and 75 outside stains) using a LightCycler 480 (Roche Diagnostics, Meylan, France) using primers 519F 5′-CAGCMGCCGCGGTAANWC-3′/907R 5′-CCGTCAATTCMTTTRAGTTT-3′ [21] and EUK345F 5′-AAGGAAGGCAGCAGGCG-3′/EUK499R 5′-CACCAGACTTGCCCTCYAAT-3′ [22], respectively. Briefly, the 16S and 18S rRNA gene reactions were carried out in 20-μl volumes containing respectively 0.6 μl (final concentration 0.3 μM) or 0.8 μl (0.4 μM) of each primer, 4 μl of PCR-grade water, 10 μl of LightCycler-DNA Master SYBR Green I master mix (Roche Applied Science), and 2 μl of sample DNA (5 ng). PCR was done with 10 min at 95 °C, followed by 40 cycles of (i) 95 °C for 15 s (16S rRNA genes) or 94 °C for 15 s (18S rRNA genes), (ii) 63 °C for 60 s (16S rRNA genes) or 60 °C for 15 s (18S rRNA genes), and (iii) 72 °C for 30 s (16S rRNA genes) or 72 °C for 15 s (18S rRNA genes). Melting curve calculation and Tm determination were done using the Tm Calling Analysis module of Light-Cycler Software v.1.5 (Roche Applied Science). To specifically quantify the relative abundance of *Ochroconis Lascauxensis*, quantitative PCRs were also performed with a slightly modified protocol of Martin-Sanchez et al. (2013): in a total volume of 20 μl, the reactions contained 10 μl of LightCycler 480 SYBR Green I Master mix (Roche) (2×), 3 μl of sterile ultrapure water, 5 μl of sample DNA (10 ng), and 1 μl of each primer (10 μM) (347F 5′-CGGGTGAACCTATCATTGAG-3′/493R 5′-TGTTGTGCAGTCTCGTAGGA-3′). These primers amplified the RNA polymerase B gene that was acknowledged to give highest resolution [23]. For each real-time PCR assay, two negative controls and a standard curve were included. The standard curve was done from DNA extracted from a cultivated *Ochroconis lascauxensis*, ranging from 10 ng to 0.01 pg of DNA.

The real-time PCR were performed on a Light Cycler 480 Instrument II (Roche). The program begun with an initial denaturation of 15 min at 95 °C, followed by 45 cycles of 15 s at 95 °C, 30 s at 58 °C, and 30 s at 72 °C. Fluorescence was measured at 72 °C. Then, melting curve was constructed by continuous heating between 60 and 95 °C. The data was analyzed with LightCycler 480 Software (v 1.5.0 Roche). All quantitative PCR were performed on duplicate for each sample. Statistical analysis of qPCR data was performed with Kruskal-Wallis tests and post-hoc Wilcoxon pairwise tests, or with ANOVA and post-hoc Tukey-HSD using the VEGAN package [24] in R 3.3.0.

Three gene markers of microbial diversity were analyzed using high throughput sequencing: the 16S rRNA genes specific for bacteria, the 18S rRNA genes specific for micro-eukaryotes (including fungi), and the second internal transcribed spacer (ITS2) specific for fungi only. This double sequencing approach targeting both eukaryotic 18S rRNA genes and ITS2 was chosen because ITS resolution is considered much higher than 18S rRNA genes for fungi [25]. For bacterial 16S rRNA gene amplifications, we used the primers 341F 5′-CCTACGGGNGGCWGCAG-3′ and 805R 5′-GACTACHVGGGTATCTAATCC-3′, which target the V3-V4 regions [26]. We used the primers 18S_0067a_deg 5′-AAGCCATGCATGYCTAAGTATMA-3′ and NSR399 5′-TCTCAGGCTCCYTCTCCGG-3′ [27] for eukaryotic 18S rRNA gene amplification, and primers ITS3_KYO2 5′-GATGAAGAACGYAGYRAA-3′ and ITS4 5′-TCCTCCGCTTATTGATATGC-3′ [28] for fungal ITS2 amplification. All PCR amplification were performed three times and pooled in equimolecular proportions prior to library construction. Libraries were built using 1 μg DNA and Illumina MiSeq sequencing of the PCR products were conducted on 2 ng DNA with specific tag sequences to concurrently sequence different samples on the same run. The amplifications and sequencing were carried out by Fasteris company (Geneva, Switzerland), using Illumina MiSeq Reagent Kit v3 (600 cycles) with paired-end mode, resulting in 2 × 300 bp sequence reads to obtain 70,000 paired reads per sample.

### Processing and analyses of sequencing data

Prokaryotic (bacterial 16S rRNA genes) and eukaryotic (18S rRNA genes and fungal ITS2) paired-end reads were demultiplexed in the different samples according to exact match to adaptors (subsequently removed). Reads presenting one or more nucleotide mismatch to adaptor or at least two mismatches with primer sequences were discarded [29]. The resulting reads were then merged using FLASh (Fast Length Adjustment of Short reads) [30] with a maximum of 10% mismatch in the overlapping region. Denoising procedures consisted in discarding reads exhibiting length outside the expected 200–500 bp range, and those containing any ambiguous bases (N). After sequence dereplication, cauterization was performed using SWARM [31], which uses a local clustering threshold rather than a global clustering threshold and an aggregation distance of 3 for identifying operational taxonomic units (OTUs). Chimeras were removed using VSEARCH and low-abundance sequences were filtered out at 0.005% (i.e., keeping OTUs representing at least 0.005% of all sequences) [32], discarding singletons from the datasets. Taxonomic affiliation of OTUs at phylum, class, genus, and/or species level was performed with RDP Classifier [33] against the SILVA database v. 132 [34] for both bacterial 16S rRNA genes and micro-eukaryotic 18S rRNA genes and the UNITE database for fungal ITS2. This procedure was automated in the FROGS pipeline [35]. Alpha diversity indices including Chao1 [36], Shannon’s H′ [37], and Simpson 1-D [38] were measured using the Paleontological Statistics (PAST) software v3.14 [39] based on OTU table and were measured for 5000, 15,054, and 15,201 randomly chosen sequences per sample for 16S rRNA genes, 18S rRNA genes, and ITS, respectively. The diversity indices were assessed with Kruskal-Wallis tests and post-hoc Wilcoxon pairwise tests, or with ANOVA and post-hoc Tukey-HSD using VEGAN package in R 3.3.0. Community structure was analyzed using a matrix that was square root-transformed to minimize the impact of highly dominant phyla or classes, and then subjected to statistical analyses to compare samples and ecological conditions. Microbial communities were primarily compared by non-metric multidimensional scaling (NMDS), and NMDS analysis of RNA data was carried out considering the most abundant OTUs (> 0.1%) only. The stress value calculated to measure the difference between the ranks on the ordination configuration and the ranks in the original similarity matrix for each replicate was considered acceptable when below 0.1 [40]. Non-parametric statistical test of analysis of similarities (ANOSIM) was conducted to test differences in overall microbial community composition in phyla or classes between different mineral substrates, seasons, or occurrences on or outside stains, and to further confirm the results observed in the NMDS plot. All analyses were based on similarity matrices calculated with the Bray-Curtis similarity index [41] and statistical analyses were performed using PAST.

### Phylogeny, networks analysis, and community comparisons using ANCOM

Common OTUs of the three mineral substrates were selected to build a phylogenetic tree using MOTHUR v.1.36.1 [42] and the Interactive Tree Of Life (iTOL) [43] was used to display, annotate, and manage the phylogenetic tree. To quantifying the community phylogenetic structure, the net relatedness index (NRI) and the nearest taxa index (NTI) were calculated as described in the Phylocom manual [44] using Qiime relatedness command [45]. To calculate the co-occurrence patterns between microbial communities in the passage, we used Spearman correlation coefficient based on work of Williams et al. [46]. Correlation values > 0.60 with *P* < 0.001 were selected. For network construction, correlation values > 0.85 were used. The network visualization was carried out by Gephi 0.9.1. The network was constructed with the spatialization of Fruchterman-Reingold, which highlights the complementarities [47]. Two statistics tests were calculated. The average weighted degree, which calculated the mean number of links for each node and the Eigenvector centrality which distinguished important nodes. Data were filtered according to the degree range, which represents the minimum number of links for each node inside the network. The filter was fixed to 5, which was the number of links displayed by 75% of the dataset. Community comparison were performed using ANCOM to distinguish significantly different OTUs between pairs of samples taken at two different dates, different substrates, or inside/outside black stains [48]. Dissimilarity distances were then calculated using R from Sørensen matrices based on presence/absence of significant OTUs in the different pairwise comparisons. Dendrograms were built using R to illustrate dissimilarity between the different pairwise comparisons. DNA and RNA communities were also compared using ANCOM to distinguish significantly different OTUs [48].

## Results

### Microbial abundances according to time, mineral substrate, and in relation to black stain presence

**Fig. 2:**
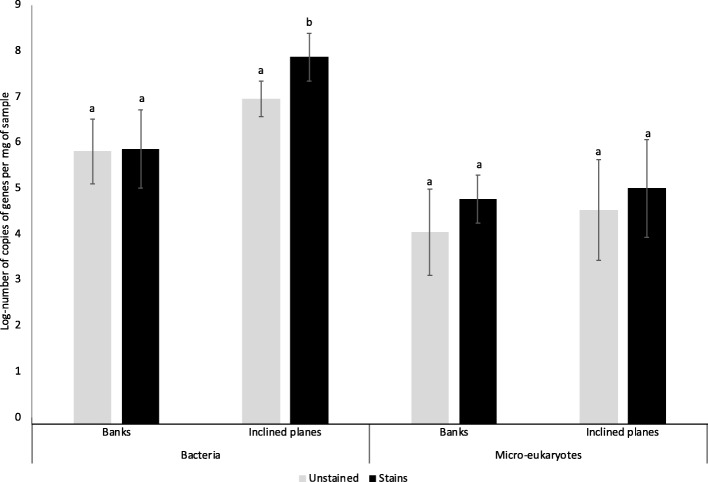
Bacterial and micro-eukaryotic abundances according to presence of black stains. Abundance data are shown as mean log number of 16S rRNA and 18S rRNA genes copies ± standard errors. Quantitative PCR analysis was performed in duplicate. Letters indicate post-hoc grouping according to significant differences between sample origin

The copy number of bacterial 16S rRNA genes varied between 4.00 • 10^7^ and 8.56 • 10^7^ copies per mg of sample. There was no significant variation of bacterial abundance between samples collected on or outside stains in banks (Wilcoxon test, *P* = 0.065) (Additional file 1: Figure S2), or in time (Wilcoxon test, *P* = 0.069) (Fig. 2a). However, bacterial abundance was significantly lower (Wilcoxon test, *P* < 2 • 10^−16^) in the banks (4.48 • 10^6^ copies per mg) than in soil and inclined planes (1.02 • 10^9^ and 9.75 •10^7^copies per mg, respectively) when considering all samples together (Fig. 2b). The number of copies of 18S rRNA genes varied between 2.53 • 10^4^ and 1.65 • 10^6^ copies per mg of sample, but it did not vary significantly according to stain presence/absence (Wilcoxon test, *P* = 0.98) (Additional file 1: Figure S2). Against this background, eukaryotic abundance was significantly lower in May–June 2016 and higher in December 2016 than at other samplings (Wilcoxon test, *P* = 6.3 • 10^−16^) (Fig. 2a), and in inclined planes (1.96 • 10^6^ copies per mg) than in soil or banks samples (9.25 • 10^5^ and 4.31 • 10^5^) (Wilcoxon test, *P* = 0.013) (Fig. 2b). There was no correlation between microbial abundance and microclimatic parameters (i.e., monthly average temperature and CO_2_ concentration prior to sampling campaign; Table 1) in the passage (Table 2).

**Table 1:** Measurements of mean temperature and CO_2_ (measured continuously from 1 month before to 1 month after the date of sampling)

	Temperature (°C)			CO_2_ (%)		
	Mean	Standard deviation	*n*	Mean	Standard deviation	*n*
Dec 2014	12.53	0.04	71	0.45	0.25	71
Jun 2015	12.59	0.03	76	1.74	0.08	77
Jan 2016	12.72	0.04	74	2.87	0.27	74
May 2016	12.73	0.03	75	1.99	0.09	77
Dec 2016	12.73	0.04	78	2.92	0.29	78

**Table 2:** Spearman correlation coefficients between microbial abundances and microclimatic conditions (measured continuously from 1 month before to 1 month after the date of sampling)

	Bacterial abundance		Micro-eukaryotic abundance	
	ρ	*P*	ρ	*P*
Temperature	0.58	0.29	0.46	0.43
CO_2_	0.78	0.12	0.71	0.17

### Variations in microbial richness and diversity indices

**Fig. 3:**
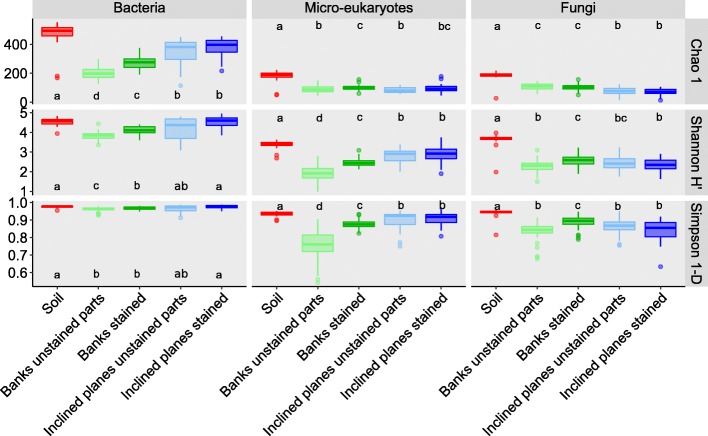
Alpha diversity indices of microbial communities according to mineral substrate and stain presence, based on estimated richness (Chao 1 index), diversity (Shannon H′ index), and evenness (Simpson index). For each index × community combination, significant differences according to mineral substrate and stain presence and post-hoc grouping are shown with letters (Wilcoxon tests, *P* < 0.05). The same findings were obtained when using the observed number of taxa instead of Chao 1 index (not shown)

For each bacterial, micro-eukaryotic, and fungal community sampled between December 2014 and December 2016, Chao1 richness index (which estimates total OTU richness) was computed from the OTU table as well as two diversity indices, i.e., Shannon H′ index (which takes into account both the numbers of individuals and of OTUs; Fig. 3) and Simpson 1-D evenness index (which represents the probability that two individuals randomly selected from a sample belong to different taxa; Fig. 3). None of the three indices showed any temporal variation for the three communities studied (Additional file 2: Figure S1), but bacterial richness/diversity indices were always higher than for micro-eukaryotes or fungi (Fig. 3).

Richness/diversity indices were also computed according to the mineral substrate and stain presence. Compared with unstained parts, Chao1 index and Shannon H′ index of bacteria on black stains did not differ on inclined planes but was statistically higher on the banks (Wilcoxon test, *P* = 3.3 • 10^−7^ and *P* = 0.0002, respectively). Both Chao1 and Shannon H′ indices were statistically higher on the soil than the inclined planes (*P* = 6.4 • 10^−6^ and *P* = 1.6 • 10^−8^, respectively) or the banks (*P* = 3.6 • 10^−7^ and *P* = 1.6 • 10^−6^, respectively). The Simpson 1-D index did not differ when considering black stains and unstained parts, both on inclined planes and banks. Simpson index on soil and inclined planes was higher than on banks (*P* = 2.9 • 10^−5^ and *P* = 0.0063, respectively).

For micro-eukaryotes, the Chao1, Shannon H′, and Simpson indices did not differ when considering black stains and unstained parts of the inclined planes, but they were statistically higher in black stains than in unstained parts of the banks (Wilcoxon test, *P* = 0.01, *P* = 0.01, and *P* = 3.1 • 10^−10^, respectively). Chao1, Shannon H′, and Simpson indices were statistically higher on soil than on banks (*P* = 1.9 • 10^−9^, *P* = 1.4 • 10^−12^, and *P* = 4.5 • 10^−12^, respectively) and inclined planes (*P* = 2.8 • 10^−9^, *P* = 3.2 • 10^−8^, and *P* = 5 • 10^−4^, respectively).

For fungi, Chao1, Shannon H′, and Simpson 1-D indices did not differ when considering black stains and unstained parts of inclined planes, whereas for the banks Shannon H′ and Simpson 1-D indices were statistically higher in black stains than in unstained parts (Wilcoxon test, *P* = 0.0003 and *P* = 1.8 • 10^−5^, respectively). Chao1, Shannon H′, and Simpson 1-D indices were statistically higher on soil than on banks (Wilcoxon test, *P* < 2 • 10^−16^, *P* = 4.7 • 10^−9^, and *P* = 3.9 • 10^−9^, respectively) and inclined planes (*P* < 2 • 10^−16^, *P* = 6.3 • 10^−7^, and *P* = 2. • 10^−6^, respectively).

In summary, several microbial diversity indices were higher for black stains than unstained parts on the banks (but not on inclined planes). Soil showed higher Chao1, Shannon H′, and Simpson 1-D indices than banks and inclined planes for the three communities.

### Differences in microbial community structures

**Fig. 4:**
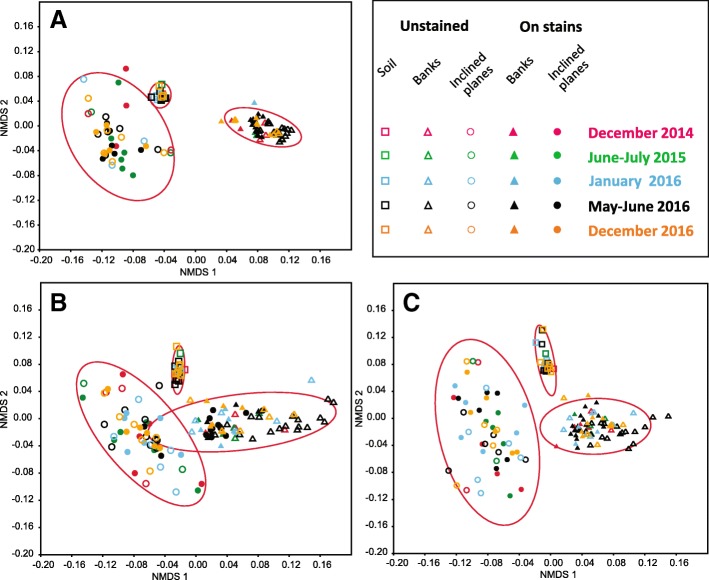
Non-metric multidimensional scaling (NMDS) analysis of microbial community structure in the passage according to time, mineral substrate and stain presence. Results are shown for bacteria (**a**), micro-eukaryotes (**b**), and fungi (**c**), and ellipses (95% confidence intervals) indicate the different mineral substrates

To compare the structure of microbial communities, non-metric multidimensional (NMDS) analyses were conducted for bacterial (Fig. 4a), micro-eukaryotic (Fig. 4b), and fungal (Fig. 4c) OTUs retrieved from DNA. Time, mineral substrate, and the presence or absence of stain were assessed as grouping parameters. For the bacterial community, NMDS distinguished three groups of samples corresponding to the three mineral substrates (stress value = 0.09, ANOSIM test, *R* = 0.91, *P* = 0.0001), whereas the effects of time and stain presence were not significant (ANOSIM test, *R* = 0.19 and 0.03, *P* = 1 and 0.03, respectively). Similarly, for micro-eukaryotes and more specifically fungi, communities were significantly different between the three substrates (stress value = 0.12, ANOSIM test, *R* = 0.70, *P* = 0.0001 and stress value = 0.16, ANOSIM test, *R* = 0.80, *P* = 0.0003, respectively) but the structure of some samples of the inclined planes and of the banks appeared similar. Time and stain presence did not structure the micro-eukaryotic (ANOSIM test, *R* = 0.10 and 0.02, *P* = 1 and 0.1, respectively) and fungal communities (ANOSIM test, *R* = 0.1 and 0.07, *P* = 0.1 and 0.001 respectively).

**Fig. 5:**
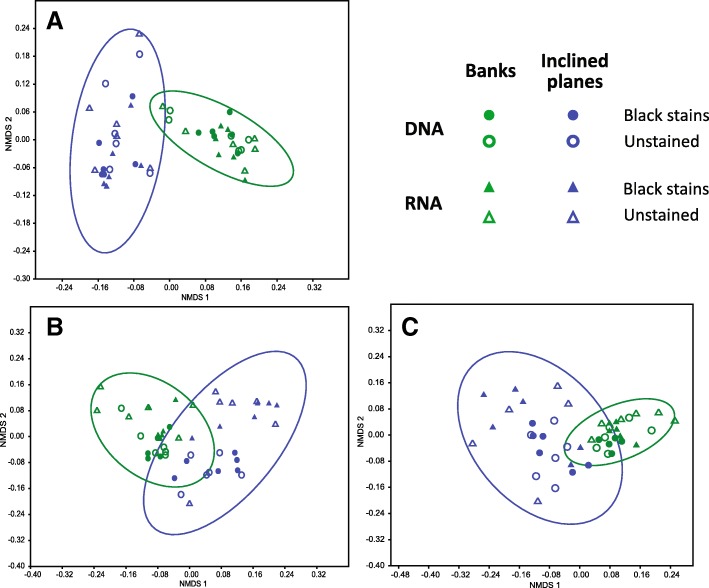
Non-metric multidimensional scaling (NMDS) analysis of the entire (DNA analysis) and transcriptionally-active (RNA analysis) microbial communities in the passage according to mineral substrate (banks and inclined planes) and stain presence. Data are shown for bacteria (**a**), all micro-eukaryotes (**b**), and only fungi (**c**). Ellipses correspond to 95% confidence intervals for each mineral substrate

Comparing community structures for bacterial, micro-eukaryotic, and fungal OTUs, obtained from RNA analysis (performed at one date for banks and inclined planes samples), allowed to assess the effects of mineral substrate and stain presence for the transcriptionally active microbial community (Fig. 5a–c). NMDS showed results similar to those observed for the entire communities, in that the effect of mineral substrate was significant but the effect of stain presence was not, regardless of whether bacteria (stress value = 0.18, ANOSIM test, *R* = 0.50, *P* = 0.0001), all micro-eukaryotes (stress value = 0.19, ANOSIM test, *R* = 0.12, *P* = 0.01), or only fungi (stress value = 0.19, ANOSIM test, *R* = 0.28, *P* = 0.0002) were considered. Nonetheless, the structure of active and entire communities showed different patterns for micro-eukaryotes on the inclined planes (ANOSIM test, *R* = 0.54, *P* = 0.001) and banks (R = 0.28, *P* = 0.006), and for fungi on the inclined planes (ANOSIM test, *R* = 0.23, *P* = 0.0078) but not on banks (ANOSIM test, *R* = 0.15, *P* = 0.08).

### Common OTUs and structuring characteristics of microbial community

**Fig. 6:**
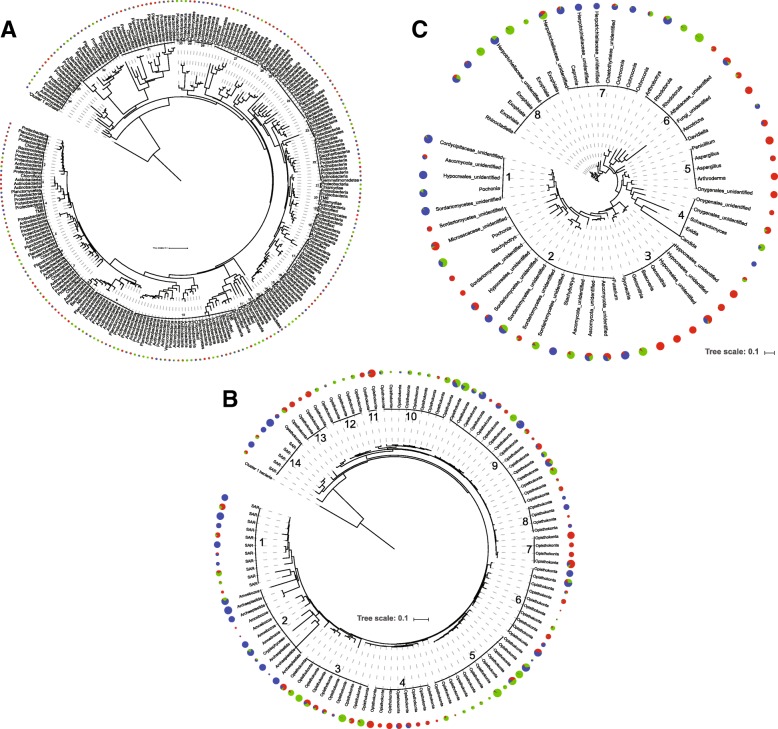
Phylogenetic tree of OTUs common to the three mineral substrates based on analysis of 16S rRNA genes (**a**), 18S rRNA genes (**b**), and fungal ITS (**c**). The size of pie charts represents the relative abundance of each OTU, and colors the distribution across mineral substrates (soil in red, banks in green, and inclined planes in blue). Groups of OTUs (i.e., groups 1–34 in **a**, 1–14 in **b**, and 1–8 in **c**) used to compute NRI and NTI values listed in Table S1 are indicated on the outside

Because mineral substrate was a parameter structuring microbial communities, we built the phylogeny of the OTUs common to the three mineral substrates to assess if these OTUs were selected by phylogeny or by environmental factors in each mineral substrate (Fig. 6). The number of common OTUs was 266 for bacteria, 113 for micro-eukaryotes (among them 88.4% were fungi), and 59 for fungi only (including black fungi *Acremonium*, *Ochroconis*, *Exophiala*, and Herpotrichiellaceae). For each of the three microbial communities, the weighted UniFrac was significantly different in all mineral substrate comparisons (Table 3), indicating that the phylogenetic structures of the common bacteria, micro-eukaryotes, and fungi was also related to mineral substrate. The occurrence of black stain was also a significant factor when considering these common OTUs (Table 3).

**Table 3:** Weighted score and significant value of UniFrac tests when comparing the effects of mineral substrates and stain presence

	Bacteria		Micro-eukaryotes		Fungi	
	W score	*P*	W score	*P*	W score	*P*
Banks vs inclined planes	0.44	< 0.001	0.53	< 0.001	0.46	< 0.001
Inclined planes vs soil	0.39	< 0.001	0.58	< 0.001	0.49	< 0.001
Banks vs soil	0.45	< 0.001	0.44	< 0.001	0.60	< 0.001
Black stains vs unstained parts	0.21	< 0.001	0.26	< 0.001	0.22	< 0.001

All samples were dominated by five bacterial phyla: the *Proteobacteria* (37.4% of all OTUs) and *Actinobacteria* (30.9%) were the most represented, followed with the three phyla *Bacteroidetes* (12.5%), *Chloroflexi* (7.0%), and *Planctomycetes* (4.9%). Micro-eukaryotes in all samples included mostly *Opisthokonta* (88.6%), a very large group that includes fungi. For fungi, the fungal classes *Sordariomycetes* and *Eurotiomycetes* were the most represented (55.5% and 28.8%, respectively) in the ITS data from all samples. One OTU corresponding to *Sordariomycetes* was abundant in the three mineral substrates. The net relatedness index (NRI) and the nearest taxa index (NTI) were calculated to quantify community phylogenetic structure and highlight the drivers of community assembly. Each index was calculated for groups of OTUs that were arbitrarily defined based on close phylogenetic relatedness (Fig. 6) and/or significant abundance in one particular mineral substrate. For bacteria, 33 of the 34 groups defined had positive indices, meaning that they tended to cluster on a phylogenetic rather than an ecological basis. For micro-eukaryotes, all 14 groups of OTUs had positive indices except the one with a high relative abundance in inclined planes samples, thus the inclined planes community tended to be overdispersed. In contrast, OTU groups of fungi with high relative abundance in inclined planes samples had high positive index values (Additional file 3: Table S1). Thus in general, the bacterial, micro-eukaryotic, and fungal OTUs common to the three mineral substrates were not phylogenetically driven by substrate types.

Comparative ANCOM analyses confirmed the importance of substrates as key drivers of community structures. Based on the presence/absence of significant clusters of OTUs, pairwise differences between substrates always branched out from the different dates or the comparisons of samples taken inside or outside stains for all clusters (Additional file 4: Figure S4), or for each microbial groups (data not shown). Two clusters were significantly different between DNA and RNA communities analyzed from bacterial 16S rRNA. Both belonged to the *Nocardia* genus, typical *Actinobacteria* from karstic caves [49].

### Specific detection of *Ochroconis lascauxensis*

We compared the relative abundance of *Ochroconis lascauxensis* either among the OTUs affiliated to this species or using a quantitative PCR approach previously developed [23]. Although these two approaches poorly correlated (Additional file 5: Figure S5), *O*. *lascauxensis* was significantly more abundant in the samples from the inclined plans compared to the two others substrates, using both quantitative PCR (Wilcoxon test, *P* < 0.001) measurements or counting the relative abundance of OTUs affiliated to this species (Wilcoxon test, *P* = 0.0128). This emblematic fungus from Lascaux cave was preponderantly found in the samples taken inside stains using qPCR approach (Wilcoxon test, *P* = 0.004) but not when considering read counts (Wilcoxon test, *P* = 0.39).

### Co-occurrence networks

**Fig. 7:**
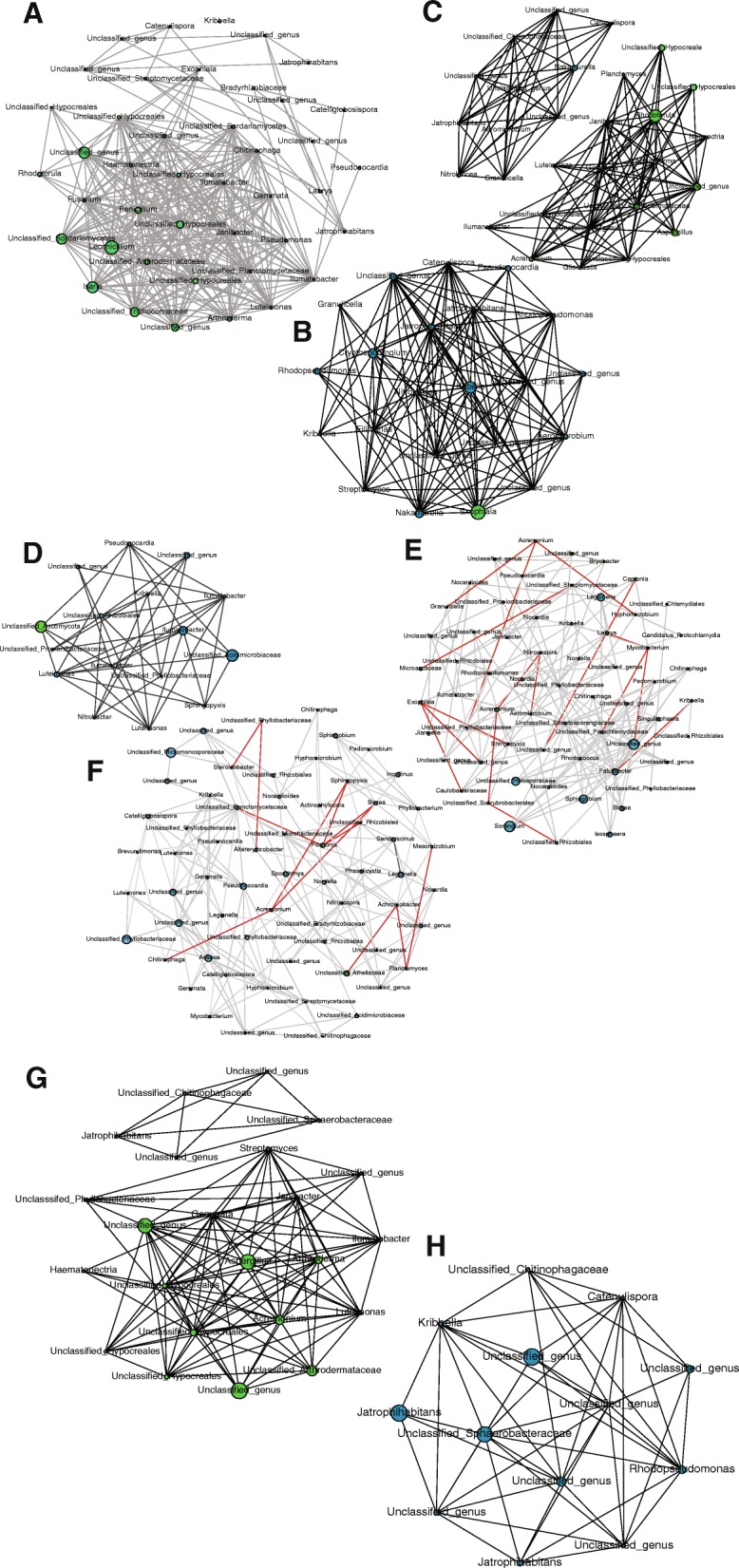
The co-occurrence networks of bacteria and fungi based on 16S rRNA and ITS MiSeq Illumina sequences. Connections materialize strong (Spearman’s ǀρǀ > 0.85) and significant (*P* < 0.001) correlations. Co-occurrence networks are shown after combining all data for December 2014 (**a**), June–July 2015 (**b**), December 2016 (**c**), soil (**d**), banks (**e**), inclined planes (**f**), unstained parts (**g**), and black stains (**h**). Blue nodes depict bacterial taxa and green nodes fungal taxa. Links in gray indicate positive co-occurrence and links in red negative co-occurrence. The size of nodes is scaled to their Eigenvector centrality

The co-occurrence of bacterial and fungal OTUs found on all three mineral substrates was represented using network analyses. These networks identified microorganisms for which sequence abundances were significantly correlated with sampling time, mineral substrate, or stain presence, which may reveal potential microbial interactions and specific consortia. Each node represented a microbial genus (bacterial in blue or fungal in green) connected by edges that were weighted by the significance of their association (positive in gray or negative in red). Regarding temporal changes, co-occurrence relations differed between the three sampling dates, i.e., December 2014, June–July 2015, and December 2016 (Fig. 7). All co-occurrence relations were positive, regardless of the date. The fungal genera with highest numbers of connections in the network were *Lecanicillium* (Fig. 7a), *Exophiala* (Fig. 7b), and *Rhodotorula* (Fig. 7c) for December 2014, June–July 2015, and December 2016, respectively. The date networks were different by their size, their composition in microorganisms, and the main nodes that built them. The networks illustrated possible interactions in different mineral substrates, and they showed different patterns (Fig. 7). In soil, all co-occurrence relations were positive. A single fungus was present in that network, and the highest number of edges was displayed by unclassified *Acidimicrobiaceae* bacteria (Fig. 7d). In the banks, 62 taxa including 5 fungi made up the network, with 157 positive and 17 negative edges. The five main nodes corresponded to bacteria (Fig. 7e). The inclined planes network had 163 positive and 12 negative edges. Four bacterial genera (*Asanoa*, *Pseudonocardia*, unclassified *Micromonosporacea*, and unclassified *Phyllobacteriaceae*) presented the highest numbers of edges, while the network included only three fungi (*Acremonium*, *Pochonia*, and unclassified *Ateliaceae*) that were noticeably negatively correlated with the network (Fig. 7f). Finally, we built networks on and outside stains. The network outside stains was composed of one group of bacteria and another, disconnected group, which contained only fungi, had the highest numbers of connections and displayed only positive co-occurrence (Fig. 7g). The network of co-occurrence on stains had only positive co-occurrence of bacterial genera (Fig. 7h). All described networks did not include the same actors, which pointed to the complexity of interactions between microorganisms according to seasons, mineral substrate, or stain presence.

## Discussion

This work focused on the passage, the central area of Lascaux Cave connecting the entrance and Hall of the Bulls to the Apse, Nave and Chamber of the Felines located deeper in the cave, and where black stains started to form in the mid 2000s [2]. Black stains represent advanced cave wall alteration processes, linked to the development of black fungi that may resist to certain toxic organic chemicals through synthesis of melanin pigments [50] while being able to catabolize the others [1]. Black stains should therefore represent very specific ecological conditions resulting from both fungal development as well as biotransformation of toxic chemicals and melanin synthesis. Indeed, Chao1 (for micro-eukaryotes), Shannon H′ (for bacteria, micro-eukaryotes and among them fungi), and Simpson indices (for micro-eukaryotes including for fungi) were statistically higher on black stains than unstained parts, for the banks (but not the inclined planes). Rejecting our hypothesis, however, the presence of black stains did not have a significant overall impact on microbial communities on the banks or inclined planes of the passage. This was indicated by comparisons targeting the bacterial community, the fungal community, and/or the micro-eukaryotic community at large, based on qPCR data of 16S and 18S rRNA genes, microbial richness and diversity indices, NMDS comparisons of microbial community structure (DNA analysis), NMDS comparisons focusing on transcriptionally active microbial communities (RNA analysis), and dissimilarity analyses of significantly different clusters using ANCOM [48]. In particular, emblematic black fungi isolated from Lascaux [50] and associated to melanin synthesis [51] were also evidenced outside of black stains and at levels similar (or sometimes higher) to those within stains. This was for instance the case for *Herpotrichiellaceae* on the banks (8.1% in stains and 2.4% outside stains) and inclined planes (11.6% in stains and 10.5% outside stains), *Exophiala moniliae* on the banks (17.9% in stains and 43.8% outside stains) and inclined planes (0.6% in stains vs 0.1% outside stains), and *Ochroconis lascauxensis* on the banks (1.8% in stains and 3.7% outside stains) and inclined planes (11.2% in stains and 6.5% outside stains). This last fungi was however significantly found more abundant in stains when using qPCR approaches. This discrepancy may indicate that despite being highly resolutive, measuring ITS read counts may not disclose the exact abundance of the different species, as compared to the *rpo*B gene targeted in our qPCR approach. Genome sequencing of the various isolates of this species could help in resolving this issue. Overall, our results suggest that precedent chemical treatments in Lascaux Cave could have selected similar types of microorganisms on different mineral substrates of the passage, independently of black stain formation. The current situation regarding black stain alterations is rather stable in the passage, but one implication of these findings is that the establishment of pigmented fungi outside stains might enable future formation of new black stains provided fungal physiology is directed toward melanin synthesis pathways. Hence, the need to avoid any further chemical treatments in the passage.

Our investigation relied on passage monitoring over 2 years, and showed that seasonal successions had little impact if any on cave microorganisms in the passage. This is in accordance with the modest climatic variations (temperature and CO_2_) that were recorded during all sampling campaigns (from December 2014 to December 2016) and the overall stability of cave wall surface quality in the passage in recent years. Indeed, climatic conditions were relatively stable through time (12.66 ± standard deviation 0.09 °C and 1.99 ± 1.00% CO_2_), even though temperatures in December 2014 and June–July 2015 were significantly lower (Wilcoxon test, *P* < 2 • 10^−16^) than at other dates (by 0.2 °C) and CO_2_ concentrations significantly higher (Wilcoxon test, *P* < 2 • 10^−16^) in January and December 2016 than in May–June 2016 (by 1%). Cave climatic conditions are often considered to be stable in time [52, 53], and when fluctuations are monitored typically they are not connected with microbiology assessments [54–56]. Martin-Sanchez et al. (2012) had also taken cave samples at different dates, over 5 years, but without resampling at similar locations, which did not allow characterizing the effect of time.

The main effect evidenced in this work was the strong impact of mineral substrates on the microbial community structure in the passage. These three mineral substrates differ in their physicochemical composition and history. In terms of chemical treatments, all three received biocides during the microbial crises [5]. While the soil was covered with quicklime, banks and inclined planes were treated with benzalkonium chloride solutions and antibiotics [3]. Noticeably, black stains occurred only once (mid 2000s) on the surface of the soil, but never came back after removal, most likely due to its composition, physical protection from benzalkonium chloride, and/or microbial particularity. The soil was also the only mineral substrate in direct contact with visitors during touristic operations, thus it potentially received organic matter such as hair, external ground traces, etc. Although not absent afterwards, organic inputs have been reduced to their minimal possible levels to limit production since the cave public closure in 1963. These various factors contributed to the differences between mineral substrates in the passage and therefore to different microbial communities. Accordingly, microbial abundance and diversity on soil was higher than on the two other mineral substrates. Similarly, Brewer et al. (2017) found that microbial community composition on tales from tombs was driven by rock type and particularly by differences in physical (porosity) or chemical (pH) characteristics. Despite the application of chemical treatments on all three mineral substrates, black pigmentation was spatially heterogeneous, resulting in patches (black stains).

As indicated by Unifrac comparison, mineral substrates strongly structured microbial community, as well as its phylogenetic structure. Nevertheless, we observed no particular phylogenetic patterns associated to the mineral substrates, as determined by measuring NRI and NTI indexes that were in most cases highly clustered. This suggests a predominance of clustering process throughout all observed taxa, which might reveal general pressure of previous antimicrobial treatments. None of the measured environmental factors seem to drive microbial community assembly, but taking samples for physicochemical analyses was not possible and only temperature and CO_2_ concentration were available. However, most pairwise interactions (as assessed by co-occurrence networks) between bacteria and fungi in these communities were positive, independently of date, mineral substrate, and stain presence. Although not causal, such positive interactions might be indicative of cooperative interactions, as oligotrophic environments imply that high-energy reactions need to be performed by cooperative microorganisms to enable growth [15]. The co-occurrence networks differed in time (Fig. 7a–c), which suggests that the participation of the microbial taxa to these networks might be optional and form temporarily according to the needs of the time, e.g., depending on resource availability or effects of chemical residues. Fungi with black pigmentation potential displayed contrasted status in relation to these networks, and in any case they did not belong to the same co-occurrence networks. *Acremonium* was found in co-occurrence networks outside stains (Fig. 7g), where it was linked only with other fungi, but *Exophiala* found in June–July 2015 network was linked only with bacteria (Fig. 7b). In contrast, *Ochroconis* did not show in any co-occurrence network, except when focusing outside stains on banks (Additional file 6: Figure S3A). This raises the possibility of contrasted ecological strategies for these fungi. Cooperation can be associated with metabolite conversion in certain cases [57] and this might be important for melanin synthesis in certain fungal taxa, but perhaps not in other taxa. Therefore, it could be that black stain formation might not require a particular consortium of microorganisms, or at least a single consortium. Rather, the advent of fungi with pigmentation potential only in co-occurrence networks outside stains when considering specific mineral substrates, i.e., *Acremonium* and *Ochroconis* on banks (Additional file 6: Figure S3A) and *Exophiala* on inclined planes (Additional file 6: Figure S3C), raises the possibility that microbial interactions with these fungi might be important to avoid melanin synthesis, and this deserves further research attention.

## Conclusion

We found that mineral substrate was an important driver structuring the bacterial, micro-eukaryotic, and fungal communities in the passage of Lascaux Cave, more so than the presence of black stains did. This study highlights the potential for multiple interactions between bacteria and fungi, and shows that a global approach on microbial communities can help better understand the conditions of stain formation. A promising perspective will be to assess the transcriptomic activities of microbial community and determine the functional profiles and metabolic pathways implicated in black stain formation.

## Additional files

Additional file 1: **Figure S2.** Bacterial and micro-eukaryotic abundances according to time (along with mean temperature and CO_2_ concentration) (A) or mineral substrate (B). Abundance data are shown as mean log number of 16S rRNA and 18S rRNA genes copies ± standard errors. Quantitative PCR analysis was performed in duplicate. The letters represented the statistical differences between histograms. (PDF 153 kb)

Additional file 2: **Figure S1.** Biodiversity of microbial communities in the passage according to sampling time. Biodiversity was considered using Chao1 richness index, Shannon H′ index and Simpson evenness index. Variations were not significant for Chao1 (*P* = 0.15) and Shannon H′ (*P =* 0.29) (ANOVA), and for Simpson 1-D (*P* > 0.05) (Wilcoxon test). (PDF 437 kb)

Additional file 3: **Table S1.** NRI and NTI values for groups of OTUs of bacteria (groups 1–34), micro-eukaryotes (groups 1–14) and fungi (groups 1–8). (ZIP 2585 kb)

Additional file 4: **Figure S4.** Dendrogram of a comparative analysis between pairs of samples harboring different archaeal, bacterial, eukaryotic (18S rRNA genes and ITS) OTUs using ANCOM [48]. The horizontal scale indicates the Euclidean distance between pairs of pairwise comparisons. (PDF 791 kb)

Additional file 5: **Figure S5.** ITS read counts affiliated to *Ochroconis lascauxensis* according to relative abundance of *rpo*B gene count assessed by quantitative PCR. Samples taken inside (crosses) or outside (circles) stains originated from banks (blue), inclined planes (red) and soil (green). Linear correlations for stained and unstained samples are indicated in dashed or plain lines, respectively. (PDF 150 kb)

Additional file 6: **Figure S3.** The co-occurrence networks of bacteria and fungi based on 16S rRNA and ITS MiSeq Illumina sequences. Connections materialize strong (Spearman’s ǀρǀ > 0.6 for banks and Spearman’s ǀρǀ > 0.75 for inclined planes) and significant (*P* < 0.001) correlations. Co-occurrence networks are shown after combining all data (December 2014, June–July 2015 and December 2016) for unstained parts (A) and black stains (B) of the banks, and for unstained parts (C) and black stains (D) of the inclined planes. Blue nodes depict bacterial taxa and green nodes fungal taxa. Links in gray indicate positive co-occurrence and links in red negative co-occurrence. The size of nodes is scaled to their Eigenvector centrality. (DOCX 17 kb)

## Abbreviations

AD: Anno domini
ANOSIM: Analysis of similarity
ANOVA: Analysis of variance
cDNA: Complementary deoxyribonucleic acid
ITS: Internal transcribed spacer
qPCR: Quantitative polymerase chain reaction
rRNA: Ribosomal ribonucleic acid
UNESCO: United Nations Educational, Scientific and Cultural Organization
