# Supplementary figures and images for: Rock substrate rather than black stain alterations drives microbial community structure in the passage of Lascaux Cave
Source: Microbiome. 2018 Dec 5;6:216. doi: 10.1186/s40168-018-0599-9 (PMC6282324; doi:10.1186/s40168-018-0599-9)

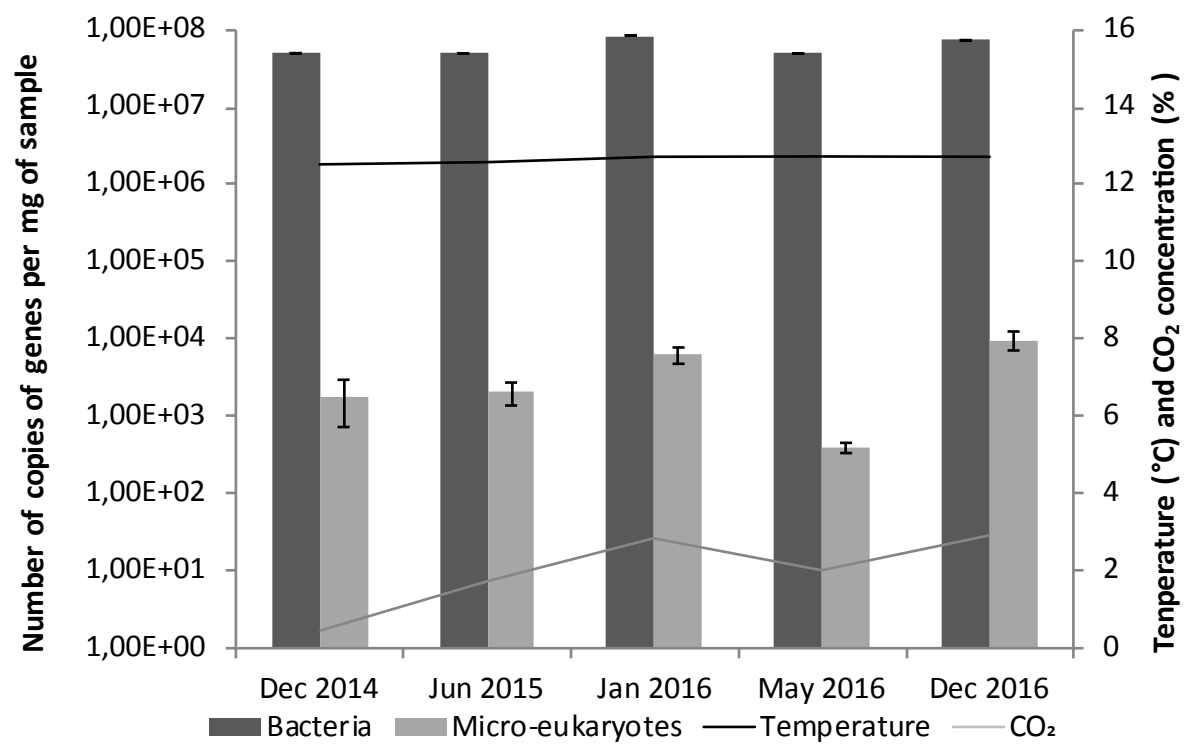

Figure S2

Supplement: Supplementary file 1 — Figure S2. Bacterial and micro-eukaryotic abundances according to time (along with mean temperature and CO2 concentration) (A) or mineral substrate (B). Abundance data are shown as mean log number of 16S rRNA and 18S rRNA genes copies ± standard errors. Quantitative PCR analysis was performed in duplicate. The letters represented the statistical differences between histograms. (PDF 153 kb) [file 40168_2018_599_MOESM1_ESM.pdf]

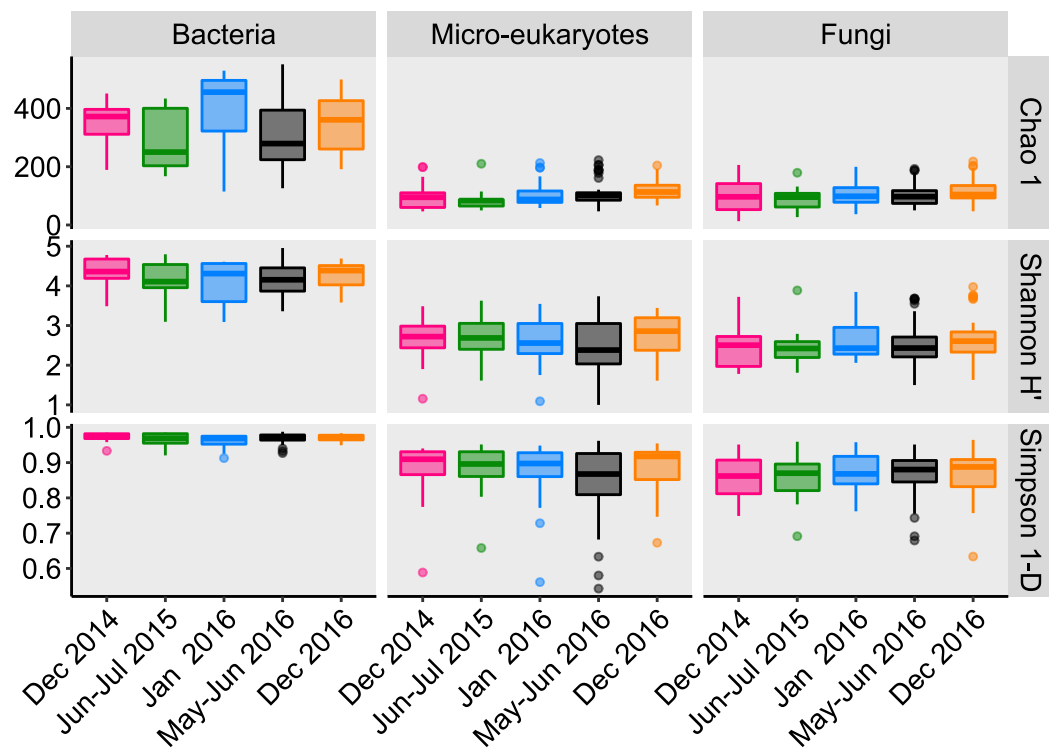

Figure S1

Supplement: Supplementary file 2 — Figure S1. Biodiversity of microbial communities in the passage according to sampling time. Biodiversity was considered using Chao1 richness index, Shannon H′ index and Simpson evenness index. Variations were not significant for Chao1 (P = 0.15) and Shannon H′ (P = 0.29) (ANOVA), and for Simpson 1-D (P > 0.05) (Wilcoxon test). (PDF 437 kb) [file 40168_2018_599_MOESM2_ESM.pdf]

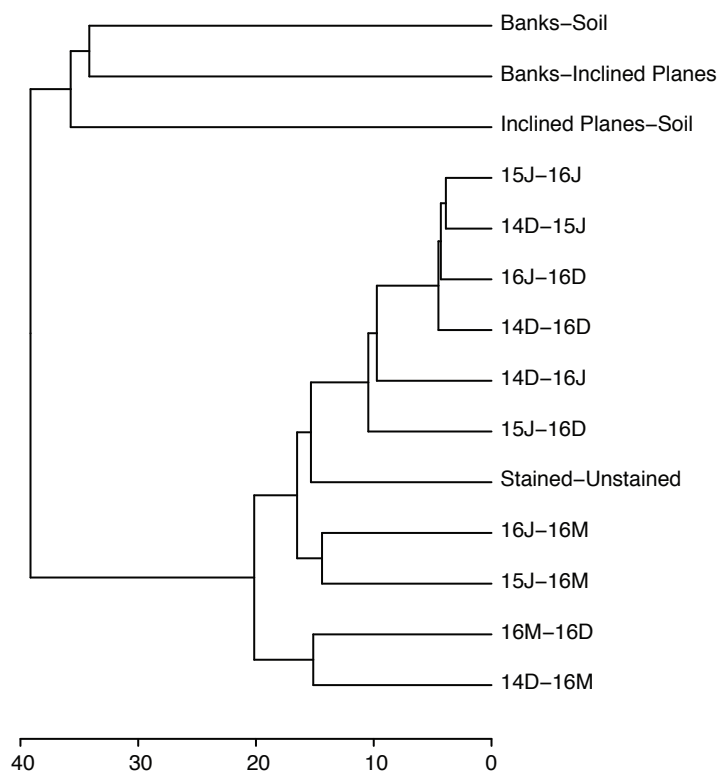

Figure S4

Supplement: Supplementary file 4 — Figure S4. Dendrogram of a comparative analysis between pairs of samples harboring different archaeal, bacterial, eukaryotic (18S rRNA genes and ITS) OTUs using ANCOM [48]. The horizontal scale indicates the Euclidean distance between pairs of pairwise comparisons. (PDF 791 kb) [file 40168_2018_599_MOESM4_ESM.pdf]

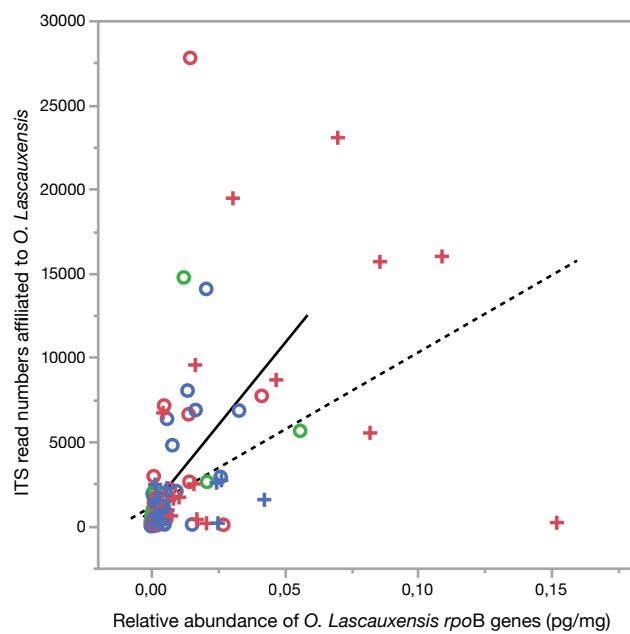

Figure S5

Supplement: Supplementary file 5 — Figure S5. ITS read counts affiliated to Ochroconis lascauxensis according to relative abundance of rpoB gene count assessed by quantitative PCR. Samples taken inside (crosses) or outside (circles) stains originated from banks (blue), inclined planes (red) and soil (green). Linear correlations for stained and unstained samples are indicated in dashed or plain lines, respectively. (PDF 150 kb) [file 40168_2018_599_MOESM5_ESM.pdf]
