# Supplementary material for: Rock substrate rather than black stain alterations drives microbial community structure in the passage of Lascaux Cave
Source: Microbiome. 2018 Dec 5;6:216. doi: 10.1186/s40168-018-0599-9 (PMC6282324; doi:10.1186/s40168-018-0599-9)
Supplement: Supplementary file 6 — Figure S3. The co-occurrence networks of bacteria and fungi based on 16S rRNA and ITS MiSeq Illumina sequences. Connections materialize strong (Spearman’s ǀρǀ > 0.6 for banks and Spearman’s ǀρǀ > 0.75 for inclined planes) and significant (P < 0.001) correlations. Co-occurrence networks are shown after combining all data (December 2014, June–July 2015 and December 2016) for unstained parts (A) and black stains (B) of the banks, and for unstained parts (C) and black stains (D) of the inclined planes. Blue nodes depict bacterial taxa and green nodes fungal taxa. Links in gray indicate positive co-occurrence and links in red negative co-occurrence. The size of nodes is scaled to their Eigenvector centrality. (DOCX 17 kb) [file 40168_2018_599_MOESM6_ESM.docx]

Table S1. NRI and NTI values for groups of OTUs of bacteria (groups 1-34), micro-eukaryotes (groups 1-14) and fungi (groups 1-8).
