# Supplementary material for: Rock substrate rather than black stain alterations drives microbial community structure in the passage of Lascaux Cave
Source: Microbiome. 2018 Dec 5;6:216. doi: 10.1186/s40168-018-0599-9 (PMC6282324; doi:10.1186/s40168-018-0599-9)
Supplement: Supplementary file 3 — Table S1. NRI and NTI values for groups of OTUs of bacteria (groups 1–34), micro-eukaryotes (groups 1–14) and fungi (groups 1–8). (ZIP 2585 kb) [file FigureS3AB.pdf › FigureS3AB.pdf]

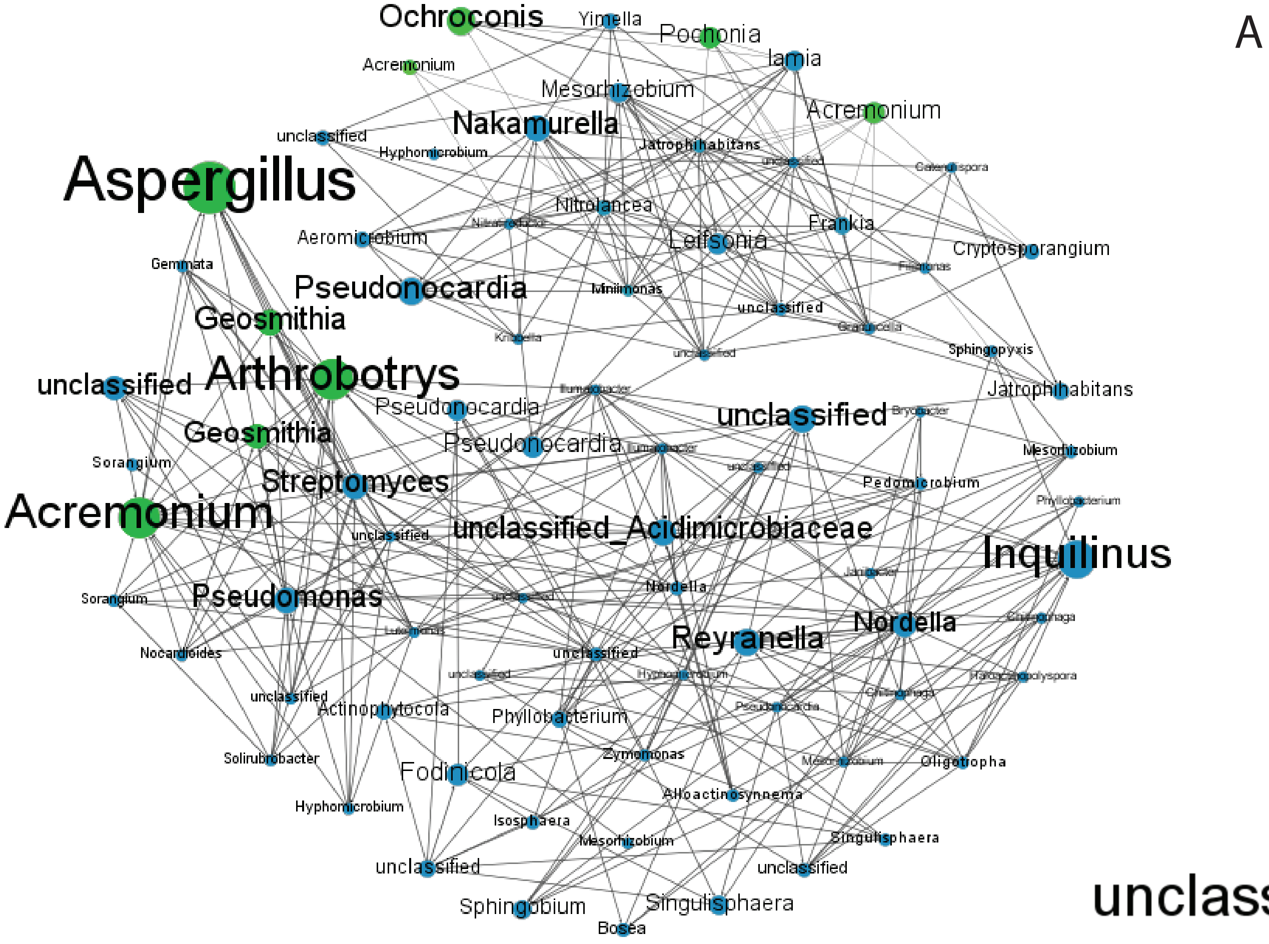
A
Ochroconis
Yimella
Pochonia
Iamia
Acremonium
Mesorhizobium
Acremonium
unclassified
Nakamurella
Hyphomicrobium
Jatrophihabitans
Aspergillus
Nitrolancea
Aeromicrobium
Leifsonia
Frankia
Cryptosporangium
Gemmata
Pseudonocardia
Minimonas
unclassified
Geosmithia
Sphingopyxis
unclassified
Arthrobotrys
Pseudonocardia
Jatrophihabitans
unclassified
Geosmithia
Pseudonocardia
Mesorhizobium
Sorangium
Streptomyces
Pedomicrobium
Acremonium
unclassified
unclassified_Acidimicrobiaceae
Inquilinus
Nordella
Sorangium
Pseudomonas
Nordella
Reyranella
Nocardioides
unclassified
unclassified
Actinophytocola
Phyllobacterium
Solirubrobacter
Zymomonas
Oligotropha
Fodinicola
Hyphomicrobium
Alloactinosynnema
Isosphaera
Singulisphaera
Mesorhizobium
unclassified
unclassified
Sphingobium
Singulisphaera
Bosea

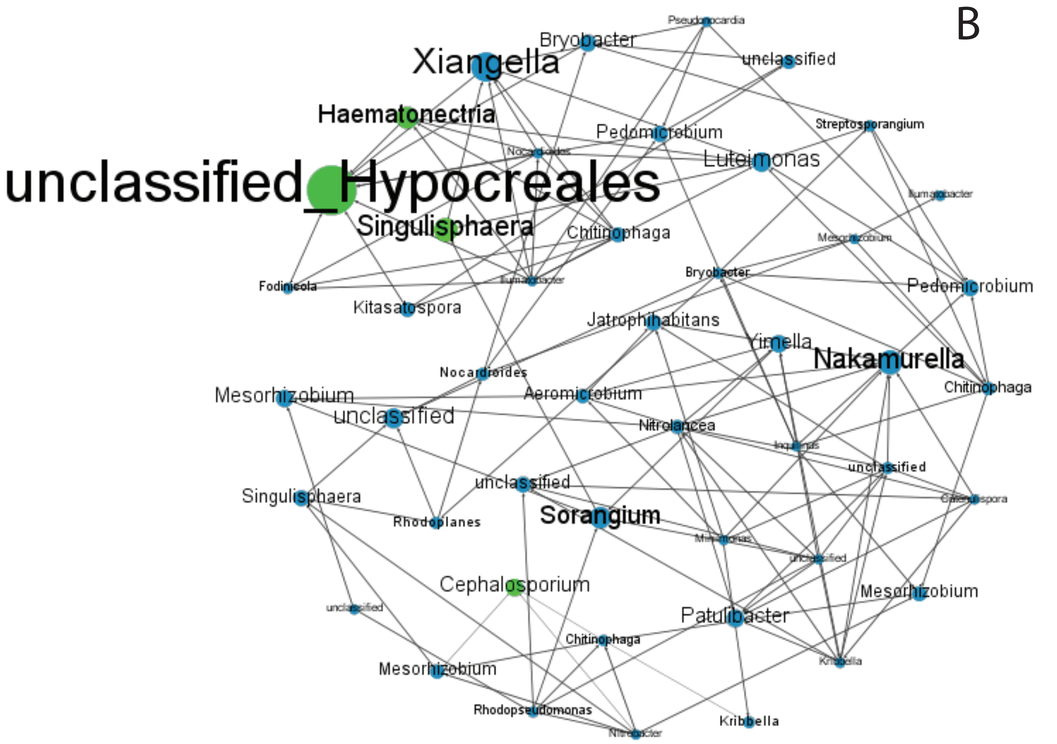
B
Bryobacter
Xiangella
unclassified
Haematonectria
Streptosporangium
Pedomicrobium
Luteimonas
unclassified_Hypocreales
Singulisphaera
Chitinophaga
Fodinicola
Bryobacter
Pedomicrobium
Kitasatospora
Jatrophihabitans
Yimella
Nakamurella
Nocardioides
Mesorhizobium
Aeromicrobium
Chitinophaga
unclassified
Nitrolancea
unclassified
unclassified
Singulisphaera
Sorangium
Rhodoplanes
Cephalosporium
Mesorhizobium
Patulibacter
Chitinophaga
Mesorhizobium
Rhodopseudomonas
Kribbella

Figure S3
