# Supplementary material for: Rock substrate rather than black stain alterations drives microbial community structure in the passage of Lascaux Cave
Source: Microbiome. 2018 Dec 5;6:216. doi: 10.1186/s40168-018-0599-9 (PMC6282324; doi:10.1186/s40168-018-0599-9)
Supplement: Supplementary file 3 — Table S1. NRI and NTI values for groups of OTUs of bacteria (groups 1–34), micro-eukaryotes (groups 1–14) and fungi (groups 1–8). (ZIP 2585 kb) [file FigureS3CD.pdf › FigureS3CD.pdf]

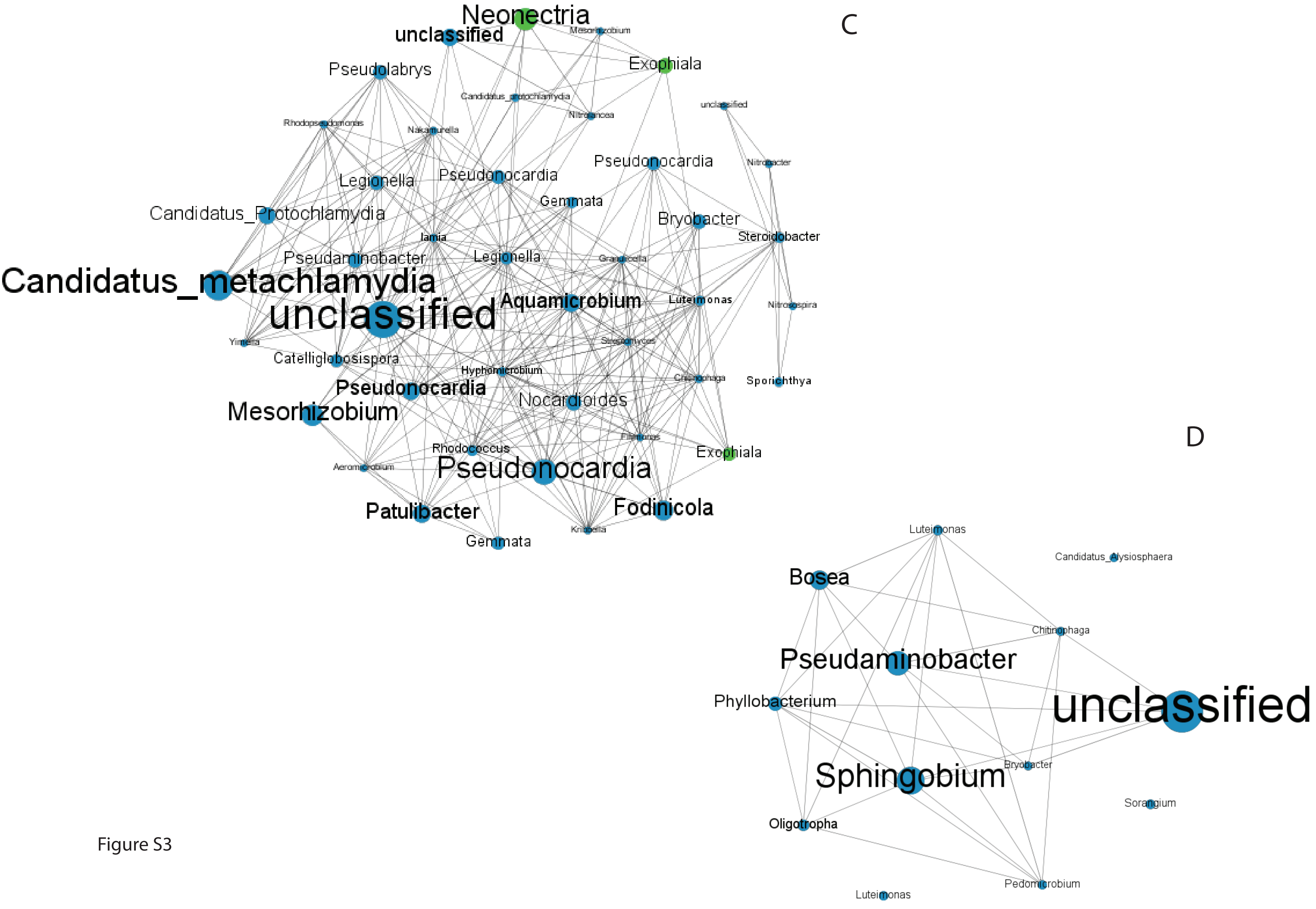
C
Neonectria
unclassified
Exophiala
Pseudolabrys
Rhodopseudomonas
Nakamurella
unclassified
Nitrobacter
Pseudonocardia
Legionella
Pseudonocardia
Gemmata
Candidatus_Protochlamydia
Bryobacter
Steroidobacter
Iamia
Pseudaminobacter
Legionella
Candidatus_metachlamydia
unclassified
Aquamicrobium
Luteimonas
Nitrosospira
Catelliglobosispora
Hyphomicrobium
Sporichthya
Pseudonocardia
Mesorhizobium
Nocardioides
Rhodococcus
Exophiala
Pseudonocardia
Patulibacter
Fodinicola
Gemmata
D
Luteimonas
Candidatus_Alysiosphaera
Bosea
Chitinophaga
Pseudaminobacter
Phyllobacterium
unclassified
Bryobacter
Sphingobium
Sorangium
Oligotropha
Pedomicrobium
Luteimonas

Figure S3
